# Genome-wide analysis suggests the importance of vascular processes and neuroinflammation in late-life antidepressant response

**DOI:** 10.1038/s41398-021-01248-3

**Published:** 2021-02-15

**Authors:** Victoria S. Marshe, Malgorzata Maciukiewicz, Anne-Christin Hauschild, Farhana Islam, Li Qin, Arun K. Tiwari, Etienne Sibille, Daniel M. Blumberger, Jordan F. Karp, Alastair J. Flint, Gustavo Turecki, Raymond W. Lam, Roumen V. Milev, Benicio N. Frey, Susan Rotzinger, Jane A. Foster, Sidney H. Kennedy, James L. Kennedy, Benoit H. Mulsant, Charles F. Reynolds, Eric J. Lenze, Daniel J. Müller

**Affiliations:** 1 Institute of Medical Science, University of Toronto, Toronto, ON Canada; 2 Campbell Family Mental Health Research Institute, Centre for Addiction and Mental Health, Toronto, ON Canada; 3 Center of Experimental Rheumatology, Department of Rheumatology, University Hospital Zurich, University of Zurich, Zurich, Switzerland; 4 Medical Bioinformatics, University of Marburg, Marburg, Germany; 5 Department of Pharmacology & Toxicology, University of Toronto, Toronto, ON Canada; 6 Department of Psychiatry, University of Toronto, Toronto, ON Canada; 7 Department of Psychiatry, University of Pittsburgh, Pittsburgh, PA USA; 8 Centre for Mental Health, University Health Network, Toronto, ON Canada; 9 McGill Group for Suicide Studies, Douglas Mental Health University Institute, McGill University, Verdun, QC Canada; 10 University of British Columbia and Vancouver Coastal Health Authority, Vancouver, BC Canada; 11 Department of Psychiatry, Queen’s University, Kingston, ON Canada; 12 Department of Psychiatry and Behavioural Neurosciences, McMaster University, Hamilton, ON Canada; 13 St. Joseph’s Healthcare Hamilton, Hamilton, ON Canada; 14 Keenan Research Centre for Biomedical Science, Li Ka Shing Knowledge Institute, St Michael’s Hospital, Toronto, ON Canada; 15 Department of Psychiatry, St Michael’s Hospital, University of Toronto, Toronto, ON Canada; 16 Healthy Mind Lab, Department of Psychiatry, Washington University, St. Louis, MO USA

**Keywords:** Pharmacogenomics, Predictive markers

## Abstract

Antidepressant outcomes in older adults with depression is poor, possibly because of comorbidities such as cerebrovascular disease. Therefore, we leveraged multiple genome-wide approaches to understand the genetic architecture of antidepressant response. Our sample included 307 older adults (≥60 years) with current major depression, treated with venlafaxine extended-release for 12 weeks. A standard genome-wide association study (GWAS) was conducted for post-treatment remission status, followed by in silico biological characterization of associated genes, as well as polygenic risk scoring for depression, neurodegenerative and cerebrovascular disease. The top-associated variants for remission status and percentage symptom improvement were *PIEZO1* rs12597726 (*OR* = 0.33 [0.21, 0.51], *p* = 1.42 × 10^−6^) and intergenic rs6916777 (*Beta* = 14.03 [8.47, 19.59], *p* = 1.25 × 10^−6^), respectively. Pathway analysis revealed significant contributions from genes involved in the ubiquitin-proteasome system, which regulates intracellular protein degradation with has implications for inflammation, as well as atherosclerotic cardiovascular disease (*n* = 25 of 190 genes, *p* = 8.03 × 10^−6^, FDR-corrected *p* = 0.01). Given the polygenicity of complex outcomes such as antidepressant response, we also explored 11 polygenic risk scores associated with risk for Alzheimer’s disease and stroke. Of the 11 scores, risk for cardioembolic stroke was the second-best predictor of non-remission, after being male (Accuracy = 0.70 [0.59, 0.79], Sensitivity = 0.72, Specificity = 0.67; *p* = 2.45 × 10^−4^). Although our findings did not reach genome-wide significance, they point to previously-implicated mechanisms and provide support for the roles of vascular and inflammatory pathways in LLD. Overall, significant enrichment of genes involved in protein degradation pathways that may be impaired, as well as the predictive capacity of risk for cardioembolic stroke, support a link between late-life depression remission and risk for vascular dysfunction.

## Introduction

Major Depressive Disorder (MDD) occurring in adults ≥60 years is frequently referred to as Late-Life Depression (LDD) and has an annual prevalence estimate of 4% in community-dwelling older adults in the United States^1^. The multidimensional interaction between ageing and depression presents a challenge in the treatment of LLD, with a significant proportion (>50%) of patients failing to achieve remission with antidepressant pharmacotherapy^2^. In particular, older adults who do not achieve LLD remission are at an increased risk of cognitive decline and dementia, possibly due to cerebrovascular disease co-occurring with depression^3^. Therefore, finding genetic markers to predict clinical outcomes may help identify novel drug targets and develop combinatorial pharmacogenomic treatment approaches and have been shown to improve depression outcomes in older adults^4^.

A growing body of evidence suggests that individual genetics contribute to antidepressant treatment outcomes and adverse drug events^5^. Genetic variability in cytochrome P450 (CYP) enzymes, which mediate the phase I oxidation of various antidepressants, has been associated with inter-individual differences in drug response and tolerability. In particular, CYP2D6, which metabolizes 50–60% of all antidepressants, appears to have the most actionable pharmacogenetic effect in older adults^6^. Although there are no specific CYP2D6 guidelines for antidepressant dosing in older adults, those with reduced enzyme function require lower doses, similarly to middle-aged adults^6^. Furthermore, age-related changes to neurotransmitter systems, such as the serotonergic system, have also been noted^7^. Although there are no actionable guidelines for the serotonin transporter gene (*SLC6A4*), a modest level of evidence suggests that older adults carrying the 5-HTTLPR low function variant (S) respond worse to antidepressant pharmacotherapy than those with high functioning variants (L/L)^6^. Although these findings are inconsistent, the observed effects of 5-HTTLPR may be similar to those in middle-aged adults, particularly of European-ancestry^8^.

Given the biological overlap between LLD, neurodegenerative disease (e.g. Alzheimer’s disease), and cerebrovascular disease, there may be common risk pathways also contributing to antidepressant non-response^9–11^. A common process overlapping these diseases is neuroinflammation. Notably, antidepressants also decrease inflammation, and conversely, anti-inflammatory treatments may alleviate depressive symptoms^12,13^. The inflammation hypothesis of depression postulates that excessive inflammatory cascades result in neurotoxicity and neuronal death in key brain regions, such as the hippocampus, contributing to depressive symptoms^14^. These effects coincide with Alzheimer’s disease neuropathology, whereby the excessive microglial response to the presence of amyloid plaques results in hippocampal atrophy^15^. Similarly, the neuroinflammatory and neurotoxic processes associated with cerebrovascular disease are observed in depressed older adults. For example, an increased burden of ischemic brain lesions (i.e. white matter hyperintensities) has been observed in neuroimaging studies of LLD^16^. Furthermore, there is an increased frequency of depression in individuals with cerebrovascular disease^11^.

This co-prevalence of cerebrovascular disease and depression supports the vascular depression hypothesis, which posits that ischemic lesions in frontostriatal regions contribute to cognitive dysfunction, depressed mood and treatment resistance^11,16^. Evidence from structural magnetic resonance imaging has shown that ischemic lesions, known as white matter hyperintensities (WHMs), are associated with and predict the onset of depression^17^. Supporting molecular evidence suggests that vulnerabilities in multiple pathways contribute to the etiology of vascular pathology, including dysregulation of the hypothalamic-pituitary-adrenal axis, endothelial function, atherosclerosis and microglial activation^11^. As such, this bidirectional relationship between vascular and neurodegeneration processes underscores the importance of the risk pathways both for LLD risk and antidepressant non-response. As such, we are interested in identifying genetic variants across enriched in vulnerable inflammatory^18^, neurodegenerative and vascular pathways, which may inform molecular targets or pathways involved in the treatment response of LLD. This project has two discovery aims. First, to describe the first genome-wide study of antidepressant response in depressed older adults. Our second aim was to present comprehensive post-GWAS analyses investigating both inflammatory and vascular pathways involved in antidepressant response and the predictive potential of polygenic risk scores.

## Methods

### Discovery cohort—IRL-GREY

Our sample consisted of adults ≥60 years from the NIH-funded clinical trial *IRL-GREY* (Incomplete Response in Late-Life Depression: Getting to Remission; NCT00892047). Participants received open-label venlafaxine (37.5 mg/day, up to 300 mg/day) for 12 weeks^19^. Inclusion criteria included a DSM-IV diagnosis of MDD with at least moderately severe symptoms as defined by a Montgomery-Åsberg Depression Rating Scale^20^ (MADRS) score ≥15. Participants with Folstein Mini-Mental State Examination (MMSE)^21^ score of <24 or DSM-IV diagnosis of dementia were exclude. In addition, individuals with unstable medical conditions, which may have required treatment with strong anti-inflammatory medications were excluded. Our final sample included 335 individuals who passed clinical and genetic data quality control (see Supplementary Figs. 1–2). The sample included 4,471,676 genotyped (Illumina PsychArray BeadChip) and imputed single-nucleotide polymorphisms (SNPs) at a 5% minor allele frequency and 99.1% call rate. All participants provided written, informed consent. The study received ethics approval from institutional review boards at the University of Pittsburgh Medical Center, Washington University School of Medicine, and Centre for Addiction and Mental Health.

### Validation cohorts

To validate any top-associated SNPs, we explored three external cohorts, which included adults treated with citalopram from Level 1 of STAR*D^22^, CANBIND-1^23^ and STOP-PD II^24^. All studies received ethics approval at relevant institutional boards and all participants provided written, informed consent. We applied the same quality control and imputation criteria to all cohorts as described for the IRL-GREY sample.

### STAR*D

We included 821 individuals of European-ancestry from Level 1 of the multi-site, clinical study STAR*D (Sequenced Treatment Alternatives to Relieve Depression; NCT00021528)^22^. Participants were diagnosed with MDD according to DSM-IV criteria and had a baseline Hamilton Depression Rating Scale (HRSD) score of ≥14. Individuals received prospective treatment with citalopram for ~8–12 weeks. Genotyping for the sample was conducted by the original authors, with approximately half the samples genotyped on the Affymetrix Human Mapping 500k Array Set and half on the Affymetrix Genome-Wide Human Array 5.0^25^.

### Canadian Biomarker Integration Network for Depression Study (CANBIND-1)

The CANBIND-1 cohort is a multicentre cohort of individuals diagnosed with MDD receiving treatment for up to 16 weeks^23^. For the first eight weeks, all participants received escitalopram (10–20 mg/day). Individuals who reached remission at eight weeks continued for another eight weeks on escitalopram, which non-remitters received augmentation with aripiprazole (2–10 mg/day). The full protocol is described in detail in previous publications. Individuals were assessed using the MADRS at nine time-points and were genotyped using the Illumina Omni 2.5 BeadChip^26^. For our investigation on antidepressant response, we used remission status from week eight of treatment before individuals received augmentation with aripiprazole.

### Sustaining Remission of Psychotic Depression II (STOP-PD II)

STOP-PD II was a 36-week randomized clinical trial (RCT) that compared the efficacy and tolerability of sertraline plus olanzapine with sertraline plus placebo in preventing relapse of remitted psychotic depression^24^. Before the RCT, patients aged 18–85 years with unipolar psychotic depression received open-label sertraline (target dose of 150–200 mg/day) and olanzapine (target dose of 15–20 mg/day) for up to 12 weeks of acute treatment. For analysis, remission of depressive symptoms was defined post-hoc as an HRSD total score ≤ 7, which was the definition used in STAR*D. Individuals were genotyped on the Illumina PsychArray BeadChip at TCAG (Toronto, Canada). We restricted the sample to 114 individuals of European-ancestry who completed the acute phase of the study with non-missing clinical (i.e. age, sex, baseline HRSD score, final HRSD score) and genotype data (i.e. European principal components and SNP genotypes).

### Genome-wide association studies

Our primary and secondary outcomes of interest were remission status defined as MADRS score ≤10 at the end of treatment (i.e. week 12)^27^ and symptom improvement defined as positive percentage change in MADRS score from baseline to end of treatment, respectively. We chose to prioritize remission as the phenotype of interest despite it being a dichotomized outcome, given that remission is considered a primary outcome of clinical antidepressant treatment^28^. We conducted genome-wide logistic and linear regressions adjusted for sex, recruitment site, age, duration of treatment, duration of the current depressive episode and the first two principal components from standard ancestry analysis (for details, see Supplementary Methods). For remission status, we also included the baseline MADRS score as a covariate.

To validate any associations, we conducted meta-analyses within three validation cohorts (i.e. STOP-PD II, CANBIND-1 and STAR*D). In the case of STAR*D and STOP-PD II, remission was defined as a Hamilton Rating Scale for Depression (HRSD) score ≤7^29^, whereas, for CANBIND-1, we used similar MADRS score ≤10 criteria as for IRL-GREY (see Supplementary Table 1). Separate associations were first conducted within each cohort, including similar covariates, were available, including sex, age, baseline depressive severity and the first two principal components from ancestry analysis. The meta-analysis was conducted using *METAL*^30^ for variants present in at least one validation cohort. Within *METAL*, variant effects were combined using an inverse-variance-weighted, average method allowing for random effects, given the heterogeneity across our four cohorts (assessed using the Cochran I^2^ test for heterogeneity). We chose to use inverse-variance weighting as opposed to weighting by the effective sample size to estimate an averaged effect size (i.e. beta). For the resulting association, we controlled for multiple testing using genomic control (i.e. lambda). Although these cohorts are heterogeneous and include younger adults, validation of our top hits would allow us to understand the generalizability of markers and their associations with venlafaxine response across the adult lifespan.

We further characterized associations using time-to-remission (Cox regression using *R* package *survival*)^31^ and response trajectories (linear mixed-effects models using *R* package *lme4*)^32^, including the same covariates (see Supplementary Figs. 3–4). Given that analyses of the smaller African, Asian-Pacific, and admixed samples would have resulted in a loss of power for genome-wide analyses, our primary analyses focused on individuals of confirmed European-ancestry.

### Genome-wide gene analyses

Genome-wide gene analyses were conducted using *MAGMA*^33^ as performed using *FUMA* v1.3.5e^34^ with default parameters (SNP-wide mean model). In brief, SNPs were assigned to genes within a 10 kb window based on a combined reference panel including 1000 Genomes Phase 3 reference panels (2504 individuals, ~84.8 million SNPs) and a subset of the UK Biobank data (10,000 individuals, ~17 million SNPs)^34^. We further explored whether top-associated genes showed over-representation for (1) tissue-specific, differentially expressed genes (DEGs), (2) known biological pathways, and (3) previous genome-wide significant associations. For gene expression, top-associated genes were compared to existing, pre-calculated DEGs across 54 *GTEx*^35^ tissues. Next, we explored geneset over-representation for biological processes, molecular functions, and cellular localization defined by Gene Ontology (10,192 genesets)^36^, as well as canonical pathways from *MsigDB*, including *KEGG*^37^, *Reactome*^38^, *BioCarta*^39^ and the *Pathway Interaction Database*^40^. Lastly, top-associated genes were compared to previously associated hits curated by the GWAS catalogue^41^. To assess over-representation, hypergeometric tests were conducted within *FUMA* with Bonferroni-correction.

### Polygenic risk scores

Polygenic risk scoring (PRS) was used to evaluate the potentially shared genetic architecture between LLD, neurodegeneration, and cerebrovascular disease. We constructed 11 risk scores for outcomes across six large genome-wide studies, including for depression^42–44^, Alzheimer’s disease^45,46^, and various strokes, such as ischemic, cardioembolic, large vessel and small vessel stroke^47^ (see Supplementary Tables). These studies were selected for their large sample sizes (i.e. mega- and meta-analyses) and including publicly available summary statistics from individuals of European-ancestry. For each outcome, scores were constructed using PRSice-2 v.2.2^48^ across ten *p*-value thresholds, with lower and upper thresholds of *P*_*T*_ = 10^−4^ and *P*_*T*_ = 1. The constructed scores were then evaluated for their association with either remission status or percentage symptom improvement using linear and logistic regressions adjusted for ancestry principal components 1 and 2, age, sex, recruitment site, treatment duration, baseline MADRS score and episode duration. To control for multiple testing, we conducted within-score permutation testing across 10,000 resamples and between-score, Bonferroni-correction for 11 scores. To control for Type I error within the process of calculating each PRS, we also conducted permutation testing to obtain an empirical p-value for the best *p*-value threshold (*P*_*0*_). Ten thousand random phenotype permutations were used to assess the PRS model under the null.

### Multiple polygenic risk scores and outcome prediction

Given the complexity of our traits of interest, we further investigated the joint predictive capacity of combining single polygenic scores, which may capture vulnerabilities across different genetic pathways. The multiple polygenic risk score (mPRS) approach has been shown to capture more genetic variance than single scores potentially^49^. Therefore, we followed a similar approach to building predictive models using elastic net regression. Elastic net regression allows for variable mixing of L_1_ and L_2_ shrinkage^50^, thereby potentially allowing for more correlation (e.g. the genetic correlation between polygenic scores) than LASSO regression, but less correlation than ridge regression (i.e. allowing for exclusion of redundant information).

First, PRS scores were calculated at a common *p*-value threshold of 0.05 for all discovery samples to avoid information leakage resulting from fitting the ‘best’ *p*-value threshold from single polygenic score analysis. After scoring individuals, we constructed the predictive models in R using the *caret*^51^ and *glmnet*^52^ packages. The cohort was randomly split into a 70% training set and a 30% holdout testing set. Within the training set, three sets of models were constructed for each outcome of interest (i.e. remission and improvement): (1) a null model with a permuted outcome; (2) a clinical model including individual, baseline predictors (i.e. sex, age, baseline MADRS score and MDE duration) and, (3) a full model including the four clinical variables and all polygenic scores. For variable pre-processing, we assessed for excessive correlation (*ρ* > 0.8) and near zero-variance (<5% frequency), as well as included dummy coding for the recruitment site. Note, separate clinical and polygenic models were not constructed to select the best predictors for a final model to avoid selection bias. Furthermore, the elastic net model includes an internal variable selection process, whereby unimportant variables (i.e. penalized coefficients = 0) are excluded from the final model.

Within the training set, alpha and lambda hyperparameters (random search grid) were tuned using 100x-repeated 10-fold cross-validation to optimize the area under the receiver operating curve (AUC) for remission, and root mean square error (RMSE) for percentage symptom improvement. The tuned models (null, clinical, and combined) were then fit on the holdout testing to assess their predictive performance. Models for remission were assessed using AUC, sensitivity, and specificity, while models for percentage improvement were assessed on RMSE, R-squared (*R*^2^), and mean absolute error (MAE). Lastly, to assess the importance of predictors, we retrieved effect sizes (i.e. beta coefficients). The significance of model performance was assessed using accuracy for remission (i.e. one-sided exact proportion test compared to null remission prevalence) and two-sided Pearson correlation test for percentage symptom improvement (i.e. predicted symptom improvement compared to observed).

**Fig. 1:**
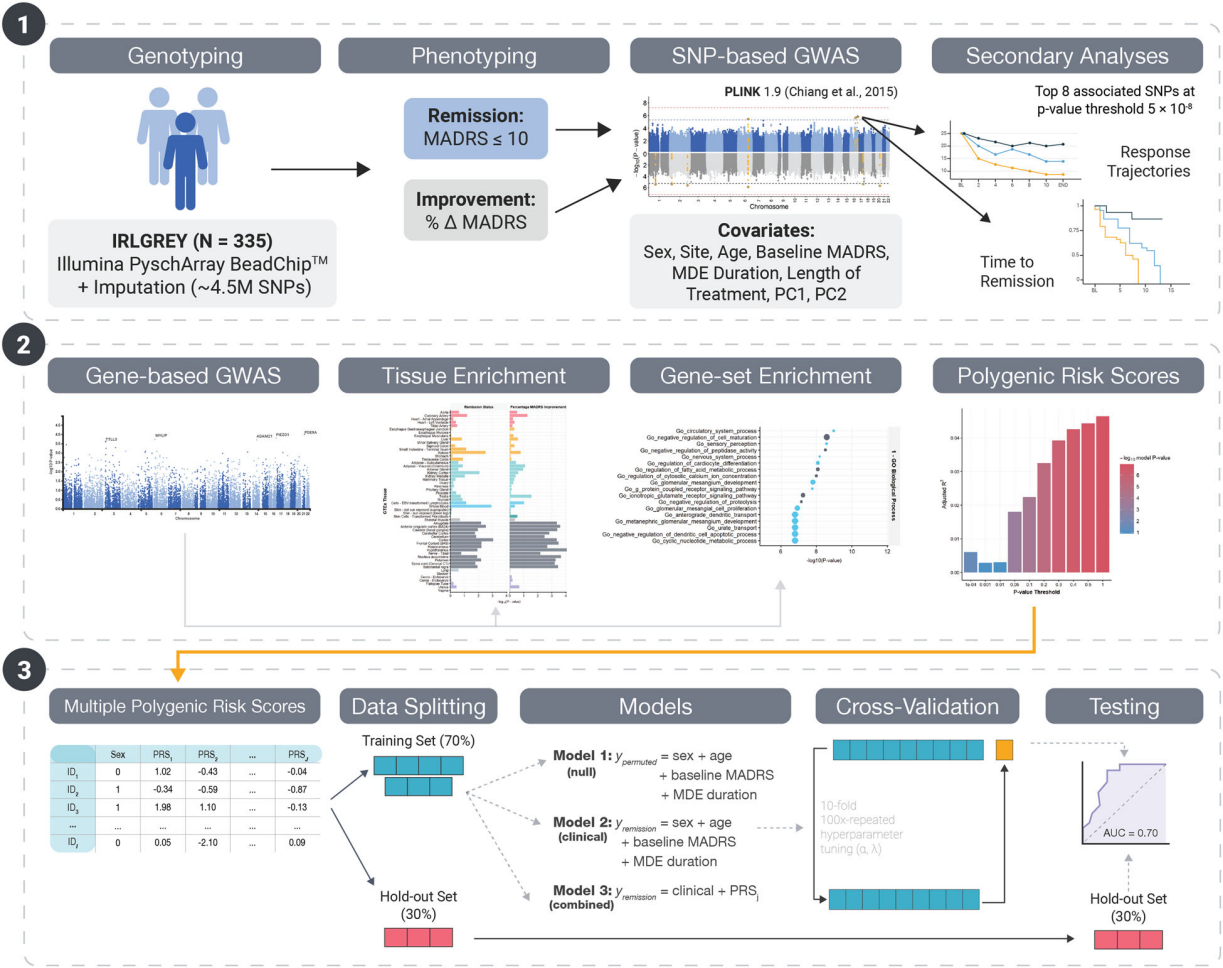
Analysis workflow. The primary presented analysis is for the IRL-GREY European-ancestry sub-cohort (*n* **=** 307). Results for the African-ancestry sub-cohort and total, mixed cohort are presented in the Supplementary Tables.

Given that IRL-GREY is a unique cohort of older adults treated with a specific antidepressant, we did not include STOP-PD, CANBIND-1, and STAR*D in the analyses. In particular, previous studies have shown that predictive models may be antidepressant-specific^53^, therefore, we do not expect that these models will generalize to older adults treated with non-SNRIs (e.g. STOP-PD II and STAR*D aged > 60) or younger adults treated with non-SNRIs (e.g. STOP-PD II aged < 60, STAR*D aged < 60, CANBIND-1). See Fig. 1 for analysis workflow overview.

## Results

### Sample demographics

Our final sample included 335 individuals who were predominantly of European (*n* = 307, 91.6%) and African (*n* = 22, 6.6%) ancestry. While the main results are presented from the European sub-cohort, where appropriate, we also present associations from the African-ancestry sub-cohort and mixed-ancestry, total sample. In the European sub-cohort, individuals were predominantly female (62.2%) with a mean age of 68.9 (*SD* = 7.0) years. In brief, 52.4% of individuals were classified as remitters at the end of treatment, reaching remission, within 10.3 weeks (*SD* = 4.7). The mean dose of venlafaxine was 241.4 mg/day (*SD* = 70.8) at the end of treatment. For additional details, including summaries for the African-ancestry sub-cohort and total, mixed-ancestry sample, see Supplementary Table 2.

### SNP-based GWAS

**Fig. 2:**
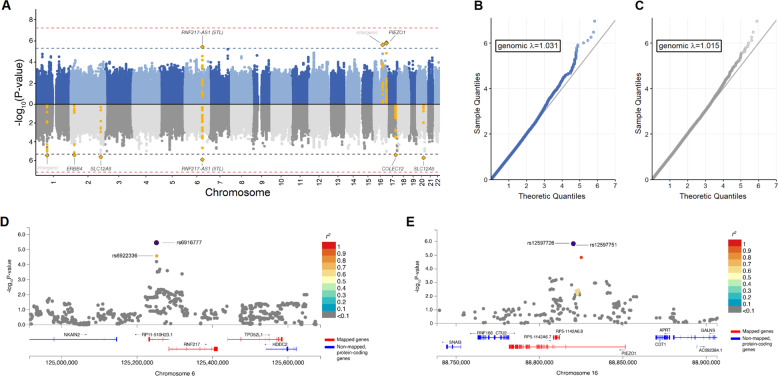
Primary analysis results. **A** Results from the SNP-based, genome-wide association study for remission status (top panel in blue) and percentage symptom improvement (bottom panel in grey). **B** Q–Q plots for association p-values with remission status. **C** Q–Q plots for association p-values with percentage symptom improvement. **D** Locus zoom plot of chromosome 6 top-associated locus surrounding rs6916777 (chr6:25251374:C:A). **E** Locus zoom plot of chromosome 16 top-associated locus surrounding rs12597726 (chr16: 88820301:G:A).

In brief, there were no genome-wide significant SNPs in association with remission status or percentage change in the MADRS score by the end of treatment (i.e. symptom improvement). However, there were eight genomic loci at a less conservative, suggestive threshold for significance (*p* = 5 × 10^−6^), which we considered of potential interest for exploration (see Fig. 2). For additional information, see Supplementary Tables 3–12.

**Fig. 3:**
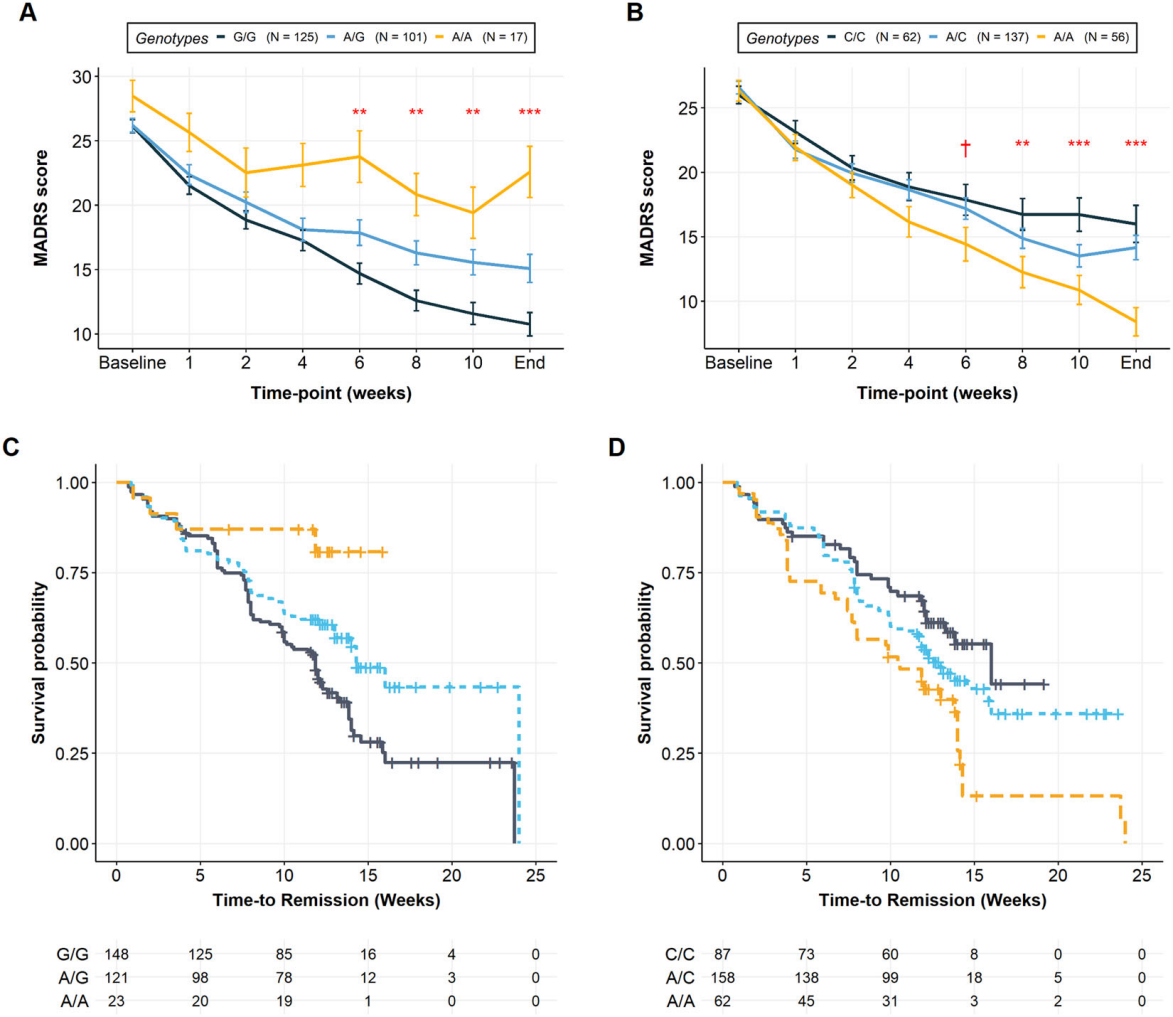
Secondary analysis results for top-associated SNPs, rs6916777 (chr6:25251374:C:A) and rs12597726 (chr16: 88820301:G:A). **A, B** Mixed-effects analyses for rs6916777 and rs12597726, respectively. Values at each time-point denote mean per genotype, and error bars denote standard error of the mean. **C, D** Kaplan–Meier survival plots for rs6916777 and rs12597726, respectively. Risk tables denote the number of non-remitted or censored individuals at each time-point. Significance levels. ***p < 0.001, **p < 0.01, *p < 0.05, ^†^*p* < 0.1.

For remission status, the top-associated variant was *PIEZO1* rs12597726 (*OR* = 0.33 [0.21, 0.51], *p* = 1.42 × 10^−6^) which also showed a non-GWAS significance in association with improvement (unstandardized beta (*B*) = −14.25 [−20.44, −8.06], *p* = 9.33 × 10^−6^). In other words, individuals carrying at least one rs12597726 effect allele (A) show a 67% decreased chance of being a remitter or 14.25% less improvement at the end of treatment. Furthermore, having at least one A-allele was associated with a worse response trajectory (*F*_*(6, 1681)*_ = 4.64, *p* = 1.06 × 10^−4^) and slower time to remission (*Median* = 14.3 weeks) compared to those with a G/G genotype (*Median* = 11.9 weeks, *95% C.I*. = [10, 13.1]) after adjusting for covariates (*HR* = 0.58 [0.42, 0.82], *p* = 1.58 × 10^−3^; see Fig. 3). For model diagnostics, see Supplementary Figs. 3–4.

Although the African-ancestry sub-cohort had a markedly low minor allele frequency for rs12597726 (3%) compared to the European-Ancestry group, in the total, mixed-ancestry sample, rs12597726 showed a similar effect (see Supplementary Table 3). Furthermore, we observed a similar directionality of association in the STOP-PD II and CANBIND-1 cohorts, which included the variant, under the random-effects models weighted both by the standard error (*OR* = 0.51, *p* = 1.48 × 10^−4^) and sample size (*N*_*eff*_ = 474.16, *Z* = −3.96, *p* = 7.46 × 10^−5^) after genomic control. Of note, the rs12597726 is an annotated regulatory feature with a predicted functionality CADD score of 12.4, suggesting that rs12597726 is among at least the 10% most deleterious substitutions in the human genome. While rs12597726 does not appear to affect PEIZO1 expression in brain tissue, rs12597726 shows the strongest effect on expression within the muscularis mucosae of the esophagus where the A-allele is associated with higher *PIEZO1* expression (*GTEx* expression, normalized effect size = 0.15, *p* = 9.4 × 10^−5^; see Supplementary Figs. 5–6).

For improvement, the top-associated variant was rs6916777 (*B* = 14.03 [8.47, 19.59], *p* = 1.25 × 10^−6^), an intronic variant in the non-coding RNA, RP11-510H23.1. Of note, rs6916777 was also one of three variants passing the suggestive threshold for remission status (*OR* = 2.58 [1.73, 3.85], *p* = 3.53 × 10^−6^). Having at least one A-allele was associated with a better response trajectory (*F*_*(6, 1676)*_ = 4.36, *p* = 1.79 × 10^−3^) and faster time to remission (Median = 13.0) compared to those with a C/C genotype (*Median* = 16.0 weeks) after adjusting for covariates (*HR* = 1.61 [1.10, 2.36], *p* = 0.01). Specifically, those with the A/A genotype reach remission at a median time of 10.4 weeks (95% C.I. = [10.4, 7.86]).

Although it is predicted that rs6916777 is unlikely to be functional (CADD *PHRED* = 0.54), carriers of the A-allele in IRL-GREY were 158% more likely to be remitters and had 14.03% greater reduction in depressive severity compared to C/C genotypes. However, we observed a significant, opposite effect of rs6916777 in the African-ancestry cohort (*B* = −26.03 [−44.75, −7.31], *p* = 0.016) despite a similar minor allele frequency. Furthermore, no effect of rs6916777 was found in the meta-analysis of the four cohorts under either weighting scheme (by the standard error, *B* = 2.22, *p* = 0.13; by sample size, *N*_*eff*_ = 1325.65, *Z* = 1.09, *p* = 0.27).

### Gene-based GWAS

Subsequently, we conducted genome-wide gene-based associations with remission status and percentage symptom improvement using *MAGMA*. The input SNPs were mapped to 17,748 protein-coding genes. No genes reached genome-wide significance after Bonferroni-correction (i.e. *α* = 0.05/17,748 genes = 2.82 × 10^−6^) for remission status or percentage symptom improvement (see Supplementary Table 13). *PDE9A* (*Z-score* = 3.71, *p* = 1.05 × 10^−4^) and *PIEZO1* (*Z* = 3.59, *p* = 1.60 × 10^−4^) were the top two genes associated with remission status, while *FPR3* (*Z* = 3.73, *p* = 9.55 × 10^−5^) and *GRIK4* (*Z* = 3.55, *p* = 1.90 × 10^−4^) were top associations with improvement. Of note, *PIEZO1* also showed a top association with improvement (*Z* = 3.48, *p* = 2.51 × 10^−4^). We saw added support for the SNP-based association for rs6916777 with *RNF217*, with *RNF217* being among the top ten genes associated with improvement (*Z* = 3.37, *p* = 3.77 × 10^−4^). In addition, we extracted 51 genes from the literature that have been previously associated with antidepressant response in MDD and LLD (see Supplementary Table 14). Across the two outcomes of interest, we observed associations of *GRIK4* as a top-hit and *SLC6A2* (*Z-score*_*Remission*_ = 2.47, *p* = 6.69 × 10^−3^, *Z-score*_*Improvement*_ = 3.55, *p* = 0.023).

### Geneset and tissue enrichment

**Fig. 4:**
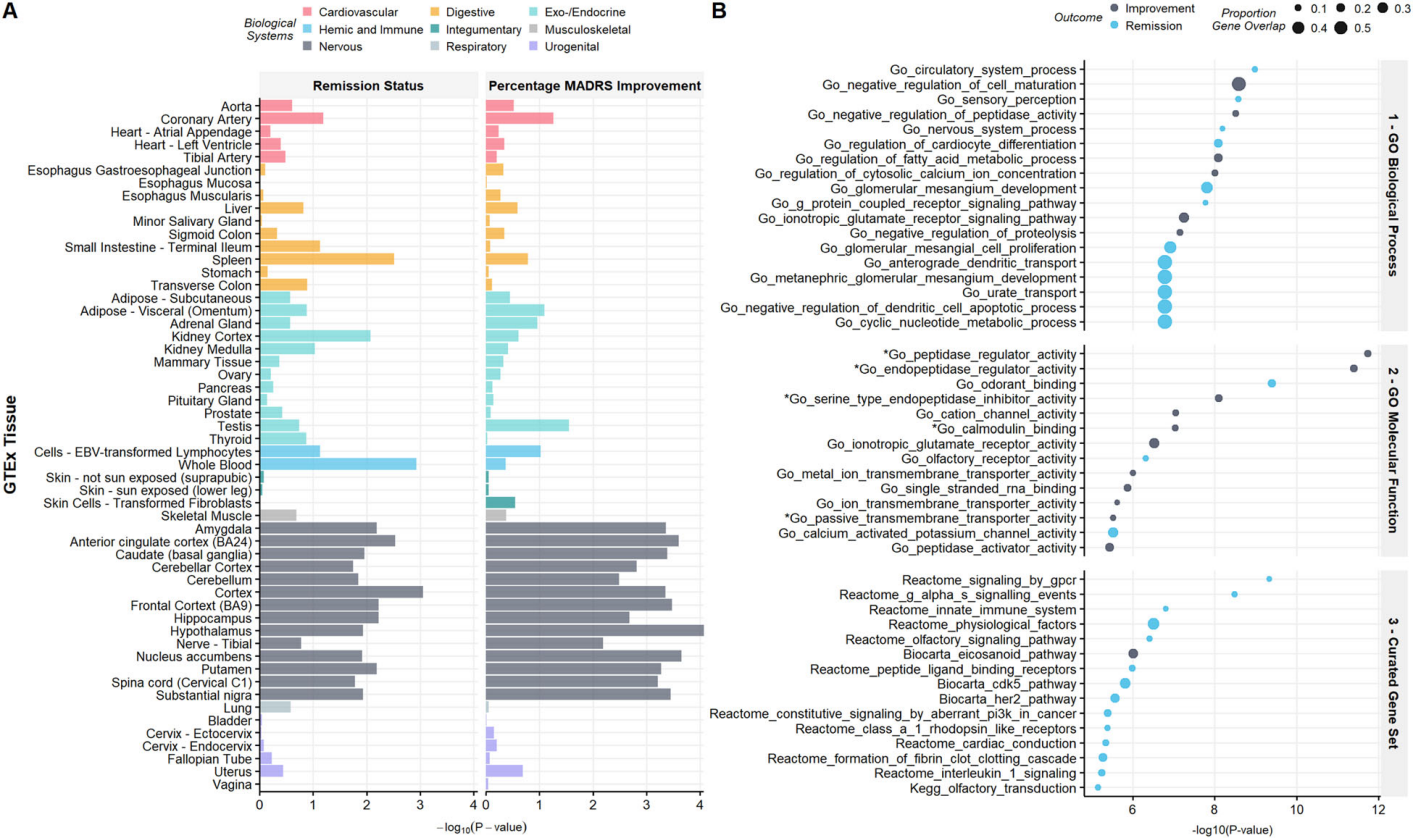
GTEx tissue and geneset enrichment for nominally associated genes with remission status (*n* = 878) or percentage symptom improvement (*n* = 889). **A** GTEx tissue (*n* **=** 54) enrichment. **B** Enrichment across Gene Ontology and curated genesets. For genesets, only the top 15 sets are shown across both remission status and percentage symptom improvement for brevity. No significant enrichment was observed for tissue or genesets. *Note. Pathways also among the top 15 associated for the second phenotype (either remission status or improvement, as indicated) but at a lower *p*-value.

We did not observe any evidence of specific tissue enrichment for remission status or symptom improvement (see Fig. 4 and Supplementary Table 15). However, we observed a significant association of two highly-overlapping pathways for peptidase regulator activity with symptom improvement (*n* = 25 of 190 genes, *p* = 8.03×10^−6^, Benjamini–Hochberg FDR-corrected *p* = 0.01). For remission status, the top-associated geneset prior to FDR correction was the GO pathway for circulatory system processes (*n* = 43 of 479 genes, *p* = 1.26 × 10^−4^, FDR-corrected *p* = 0.51; see Supplementary Tables 16–17). In addition, the top-associated GWAS Catalog genesets were for cardiac structure and function (*n* = 4 of 6 genes, *p* = 8.30 × 10^−5^, FDR-corrected *p* = 0.15; see Supplementary Table 18).

### Polygenic risk scores

**Fig. 5:**
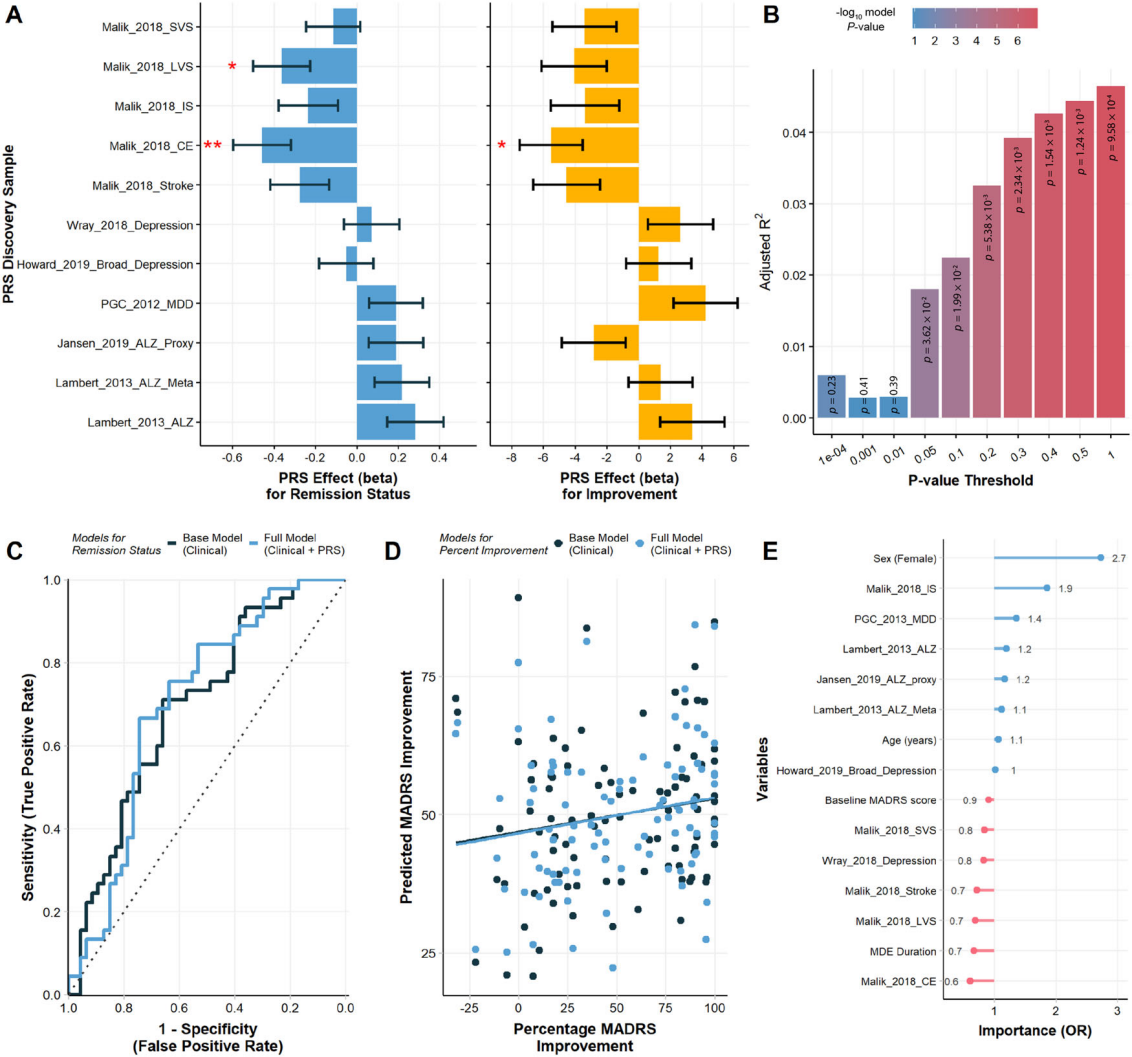
Polygenic risk scores and prediction. **A** Polygenic risk score effect sizes for remission status (blue, odds ratios) and percentage symptom improvement (yellow, unstandardized betas). Error bars denote standard error of the mean. **B** Variance in remission status explained by the polygenic risk score regression results for cardioembolic risk (Malik et al., 2018). **C, D** Receiver operating curves for prediction of **C** remission status and **D** symptom improvement comparing a base clinical model to a model including the 11 polygenic risk scores. **E** Variable importance from the best performing model for remission status.

The PRS for cardioembolic stroke was significantly associated with remission status and symptom improvement, which remained significant after permutation testing and Bonferroni-correction (*α* = 0.0045) for the 11 PRS scores that were built (see Fig. 5 and Supplementary Tables 19–20). The cardioembolic stroke PRS reached significance at a *p*-value threshold of 0.05 and achieved the highest explained variance (adjusted *R*^*2*^ = 0.046) at the most lenient *p*-value threshold, including 75,508 SNPs. In a full model, a 1 SD increase in polygenic risk for cardioembolic stroke, was associated with decreased probability of remission (*OR* = 0.63 [0.48, 0.83], *p* = 0.001, permutation *p* = 0.006) or 5.51% less improvement (MADRS, *beta* = −5.51 [−9.45, −1.57], *p* = 0.01, permutation *p* = 0.033). Overall, the addition of polygenic risk for cardioembolic stroke improved the model fit for remission as compared to a model with only clinical variables (likelihood ratio test, *χ*^*2*^_*(1)*_ = 1.21, *p* = 4.78 × 10^−4^), as well as marginally for improvement (*χ*^*2*^_*(1)*_ = 3.78, *p* = 0.052).

### Predicting treatment outcomes using mPRS

Given the association of the polygenic risk for cardioembolic stroke with treatment non-remission and less symptom improvement, we evaluated the predictive capacity of the 11 risk scores when added to a clinical model. For remission status, both models showed significant predictive performance with the mPRS model achieving marginally better performance (*AUC* = 0.70, *Sensitivity* = 0.72, *Specificity* = 0.67; *Accuracy* = 0.70 [0.59, 0.79], *p* = 2.45 × 10^−4^) compared to the clinical model (*AUC* = 0.70, *Sensitivity* = 0.64, *Specificity* = 0.71; *Accuracy* = 0.67 [0.57, 0.77], *p* = 1.13 × 10^−3^; see Supplementary Tables 21–22). In the full model, the top five predictors included sex (100% importance), risk for ischemic stroke (60.9% importance compared to sex), risk for cardioembolic stroke (48.9%), MDE duration (38%) and risk for large vessel stroke (30.8%; see Fig. 5). However, for percentage improvement, neither the mPRS model (*RMSE* = 36.93, *R*^*2*^ = 0.04, *MAE* = 31.20; Pearson’s *ρ* = 0.19 [−0.02, 0.36], *p* = 0.07) nor the clinical model (*RMSE* = 37.43, *R*^*2*^ = 0.03, *MAE* = 31.73; Pearson’s *ρ* = 0.16 [−0.04, 0.36], *p* = 0.12) was significant.

## Discussion

This is the first genome-wide study of venlafaxine response in older adults, with our sample being the largest cohort of LLD patients with genome-wide SNP and clinical data. Although we did not observe any genome-wide significant association with remission status or percentage symptom improvement, it remains important to evaluate suggestive findings in the context of previous studies to begin generating further hypotheses for investigation and to explore if already-known processes are involved in response.

Our top association with remission status was rs12597726 in the *PIEZO1* gene, which encodes for *Piezo Type Mechanosensitive Ion Channel Component 1*. PIEZO1 channels are critical for vascular remodelling, including angiogenesis, and have implications for hypertension, aneurysms and stroke^54–57^. These channels prevent microglial activation by pro-inflammatory cytokines and chemokines, including IL-1β and TNF-α, in response to neurodegenerative amyloid-beta plaques and ischemic events^58–61^. Despite generally low PIEZO1 expression levels in the brain, the rs12597726 A-allele is associated with lower PIEZO1 expression, which may ultimately contribute to higher inflammation and non-response (see Supplementary Figs. 5–6). Overall, our finding that increased vascular risk is associated with worse response to venlafaxine in our sample support implicated mechanisms and the vascular depression hypothesis^11^.

Our findings also implicate the ubiquitin-proteasome system, which is involved in intracellular protein degradation. Specifically, we observed that the intergenic variant rs6916777 showed the strongest association with symptom improvement. Although intergenic, rs6916777 is a cis-eQTL for the downstream gene *RNF217* (Ring Finger Protein 217) across multiple tissues (see Supplementary Figs. 7–8). Although the function of RNF217 remains unclear, RNF217 facilitates B-cell maintenance processes, primarily apoptosis through the ubiquitin-proteasome system^62^. In the IRL-GREY cohort, the rs6916777 A-allele was associated with better venlafaxine response, which may be due to lower expression of *RNF217*. This finding supports previous evidence of an association between the ubiquitin-proteasome system and antidepressant response^63^.

For the gene-based association study, there were no genome-wide significant genes; however, we observed whole-gene associations which echoed single-SNP results. PIEZO1 showed a nominal association, as well as *RNF217*. Other top gene associations included *PDE9A* for remission, as well as *FPR3* and *GRIK4* for symptom improvement. *GRIK4* encodes for the *Kainate-type Ionotropic Glutamatergic Receptor Subunit 4*, which has been associated with citalopram response in the STAR*D cohort^64^. Similarly, variants in the gene encoding for the *Phosphodiesterase 9* *A* enzyme (*PDE9A)* have been associated with MDD risk but not response across various antidepressants, including fluoxetine, desipramine, and citalopram^65,66^. GRIK4 and PDE9A contribute to critical pathways involved in depression and antidepressant response, including glutamatergic signalling, neuroplasticity and neurogenesis^67^. Unlike GRIK4 and PDE9A, the function of FPR3 remains unclear but shares 83% of its sequence with FPR2, which has been implicated in neuroinflammation via microglial activation^68,69^. Nonetheless, among these proteins, PDE9A is a druggable-target, which is currently in clinical trials for Alzheimer’s disease, while FPRs have been postulated as possible targets for mitigating ischemia-induced inflammation^70,71^.

In addition, we explored the polygenic overlaps between venlafaxine response in late-life, all-age depression, Alzheimer’s disease and stroke. While we observed several nominal associations, only the polygenic overlap between venlafaxine non-response and increased risk for cardioembolic and large vessel stroke remained significant. Although it is difficult to disentangle the genetic effects critical to confluent pathways involved in depression, neurodegeneration and cerebrovascular disease, further investigation is required^3^.

The main limitation of this investigation is the relatively small sample size. Given that we selected the dichotomized variable- remission as our outcome, the analysis suffered a loss of power, which may have led to a lack of significantly associated variants. Therefore, we attempted to validate any putative associations in three external cohorts of adults treated with antidepressants. However, these cohorts were heterogeneous in age and venlafaxine treatment, which did not allow for replication. Notably, the validation cohorts included treatment with SSRIs that may have different pharmacogenetic contributions than venlafaxine, a dual serotonin-norepinephrine reuptake inhibitor. While we also observed a significant predictive effect of polygenic risk scores for cardioembolic and large vessel stroke, overall, the improvement in performance compared to a base clinical model was minimal. However, due to the polygenicity of a complex outcome such as antidepressant response, variants likely contribute small effects and the addition of other risk scores, for example, for other psychiatric, neurodegenerative and cardiovascular outcomes, may improve performance.

In the context of existing literature, our results underline the importance of neuroinflammation, as well as vascular health and its consideration in venlafaxine treatment in older adults. Among the genes identified, such as PDE9A, there is a potential to inform druggable targets and further exploration of drug repurposing and treatment development to address neuroinflammatory and vascular pathways. However, further investigations will require more extensive and pooled samples to increase the power to detect non-spurious associations with small effects. Overall, these findings elucidate contributions to venlafaxine treatment response, particularly in older adults who may have unique pharmacokinetic and pharmacodynamic characteristics.

## Supplementary information

Supplementary Methods

Supplementary Figures

Supplementary Tables
