# Supplementary material for: Genome-wide analysis suggests the importance of vascular processes and neuroinflammation in late-life antidepressant response
Source: Transl Psychiatry. 2021 Feb 15;11:127. doi: 10.1038/s41398-021-01248-3 (PMC7884410; doi:10.1038/s41398-021-01248-3)
Supplement: Supplementary file 1 — Supplementary Methods [file 41398_2021_1248_MOESM1_ESM.pdf]

Supplementary Methods

Genome-wide analysis suggests the importance of vascular processes and neuroinflammation in late-life antidepressant response

1. Quality Control

1.1. Genotyping and Quality Control

Endorsed by the Psychiatric Genomics Consortium, the PsychArray contains 265,000 tag SNPs from the Illumina HumanCore BeadChip, 245,000 markers from the HumanExome BeadChip and 50,000 SNPs which have previously been associated with common psychiatric disorders.

From our initial sample of 453 patients, we excluded 91 individuals who were withdrawn for several reasons (see Supplementary Figure 1). Thirteen individuals were removed due to excessive heterozygosity based on an inbreeding coefficient greater than two standard deviations from the sample mean. We checked individuals for discordance between self-reported and genetic sex, during which one individual was excluded due to Y-chromosome abnormalities. Lastly, one individual showed excessive relatedness ($\hat{\pi} > 0.185$, i.e., second cousins), and two individuals had excessive missing genotypes (more than 10%). Overall, we excluded 17 individuals who failed genetic quality control based on one or more criteria, resulting in a final sample of 345 individuals who entered imputation.

Genetic ancestry was assessed using multidimensionality scaling in *PLINK* v.1.9.[1] First, we pruned SNPs based on linkage disequilibrium (LD) using a 50 SNP window and shifting by five SNPs with an r^2 threshold of 0.2. We also removed predefined high-LD regions.[2] Outliers were defined as individuals' principal components 1 and 2 loadings beyond the six standard deviations from the centre of the ancestral cluster.[3] Divergent individuals (i.e., discrepant between self-reported and genetic ancestry) that were visibly clustering well with other continental populations were reclassified appropriately (e.g., African-ancestry vs. European-ancestry, N=2); however, those with ambiguous ancestries were reclassified as "admixed" (N=5, see Supplementary Figures).

1.2. Imputation

Per marker, we exclude variants based on violations of Hardy-Weinberg equilibrium at $p < 10^{-7}$, low genotyping call rate < 95%, and low minor allele frequency < 1%. Whole-genome imputation was conducted using the *genipe* pipeline,[4] which uses *IMPUTE2* v2.2[5] in 5-Mb segments per chromosome after pre-phasing with *SHAPEIT2*[6] and the *1000 Genomes* reference panel (Phase 3).[7] We filtered for biallelic SNPs and retained those with an imputation score 0.7, completion rate > 90%, and a minor allele frequency ≥ 5%. Hard genotype calls were made using a probability threshold of 90%. We included 4,471,676 bi-allelic variants with a genotyping rate of 99.1% in 329 individuals (307 European-ancestry, 22 African-ancestry).

2. Secondary Analyses

2.1. Linear mixed-effects models

For top-associated variants, we constructed linear mixed-effects models across the eight time-points of treatment. Our outcome of interest was the MADRS score at the end of treatment (week 12). We included fixed effects from age, sex, current depressive episode duration, baseline MADRS score, and additive SNP genotype (i.e., 0, 1, 2), as well as random effects from individual ID and site of recruitment (denoted below by "|").

$$y_{MADRS\ end} = \beta_0 + \beta_1 age + \beta_2 sex(female) + \beta_3 MDE\ duration + \beta_4 MADRS\ baseline + \beta_5 time + \beta_6 SNP_{genotype} + \beta_7 time{:}SNP_{genotype} + \beta_8(1|ID) + \beta_9(1|Site_1) + \beta_{10}(1|Site_2)$$

For each SNP model, we checked assumptions of homoscedasticity, normality, and linearity, as well as the presence of influential observations (see Supplementary Figures). Although we did not observe any violation of model assumptions, we observed three influential observations at weeks 2, 6, and 10 that, when removed, changed the standard error by 13%, 22%, and 20%, respectively.

2.2. Time-to remission analysis

First, we obtained the Kaplan-Meier survival curve for the entire population, as well as stratified by the SNP. We used time-to remission as our 'failure event' and assumed that censoring time is independent of failure time. The estimated survival curves across SNP genotypes or alleles were compared curves using a Mantel-Haenszel, χ^2 test with one degree of freedom at an α=0.05. Subsequently, to assess the effects of baseline covariates on time-to remission, we fit a Cox proportional hazards regression. For this, we fit two models:

$$\textit{Model 1:}\ \ \lambda_x(t) = \lambda_0(t)e^{\beta_1 age + \ + \beta_2 sex(female) + \ \beta_3 Site1 + \ \beta_4 sex(Site2) + \beta_5 MDE\ duration + \ \beta_6 MADRS\ baseline + \beta_7 PC1 + \beta_8 PC2}$$

$$\textit{Model 2:}\ \ \lambda_x(t) = \lambda_0(t)e^{\beta_1 age + \ + \beta_2 sex(female) + \ \beta_3 Site1 + \ \beta_4 sex(Site2) + \beta_5 MDE\ duration + \ \beta_6 MADRS\ baseline + \beta_7 PC1 + \beta_8 PC2 + \beta_9 SNP}$$

These two models were then compared using a likelihood ratio test (LRT, α=0.05) to assess whether the inclusion additive SNP genotypes in estimating the survival curves improves model fit. For the final Cox model (i.e., Model 2), the overall model significance was assessed using an LRT compared to a model only including the intercept. Also, ANOVA was used to assess the significance of the individual coefficients.

Given that the Cox regression assumes proportional hazards over time for validity, this assumption was tested for all predictors in the model by assessing predictor interaction with time. We obtained the Pearson product-moment correlation (ρ) between the scaled Schoenfeld residuals and log(time) for all variables, including additive SNP genotype. In addition, we also considered the global test for all interactions. These tests were considered at α=0.05, which would indicate violators of the proportionality assumption. In addition, we plotted the scaled Schoenfeld residuals against transformed time with a smoothed line, including ± two standard deviations to inspect any non-proportional effects in the model across the exposure and covariates. Any observed systematic deviations from a horizontal line were interpreted as an indication of non-proportional hazards. Lastly, we explored model outlier using deviance residuals (±3 standard deviations) given their assumed normality.

Overall, the model for rs6916777 did not violate the assumption of proportional hazards based on the global Schoenfeld test ($\chi^2_{(10)}$=11.58, *p*=0.31). For rs12597726, the global Schoenfeld test showed a significant deviations from proportional hazards ($\chi^2_{(10)}$ =18.79, *p*=0.043) due to the violations of baseline MADRS score ($\chi^2_{(1)}$=6.4, *p*=0.01) and rs12597726 genotypes ($\chi^2_{(2)}$=6.82, *p*=0.03). However, upon closer graphical inspection, there appeared to be no observable issues with non-proportionality (see Supplementary Figures).

REFERENCES

1 Chang CC *et al*. Second-generation PLINK: rising to the challenge of larger and richer datasets. *GigaScience* 2015; **4**: 7.

2 Wuttke M. Scripts for GWAS association and metaanalysis (high-LD-regions_hg19.txt). GitHub. https://github.com/genepi-freiburg/gwas.

3 Price AL *et al*. Principal components analysis corrects for stratification in genome-wide association studies. *Nat Genet* 2006; **38**: 904–909.

4 Lemieux Perreault LP, Legault MA, Asselin G, Dubé MP. genipe: an automated genome-wide imputation pipeline with automatic reporting and statistical tools. *Bioinformatics* 2016; **32**: 3661–3663.

5 Howie B, Fuchsberger C, Stephens M, Marchini J, Abecasis GR. Fast and accurate genotype imputation in genome-wide association studies through pre-phasing. *Nat Genet* 2012; **44**: 955.

6 O'Connell J *et al.* A General Approach for Haplotype Phasing across the Full Spectrum of Relatedness. *PLOS Genet* 2014; **10**: e1004234.

7 1000 Genomes Project Consortium *et al.* A global reference for human genetic variation. *Nature* 2015; **526**: 68–74.
