# Supplementary material for: Genome-wide analysis suggests the importance of vascular processes and neuroinflammation in late-life antidepressant response
Source: Transl Psychiatry. 2021 Feb 15;11:127. doi: 10.1038/s41398-021-01248-3 (PMC7884410; doi:10.1038/s41398-021-01248-3)
Supplement: Supplementary file 2 — Supplementary Figures [file 41398_2021_1248_MOESM2_ESM.pdf]

Supplementary Figures

Genome-wide analysis suggests the importance of vascular processes and neuroinflammation in late-life antidepressant response

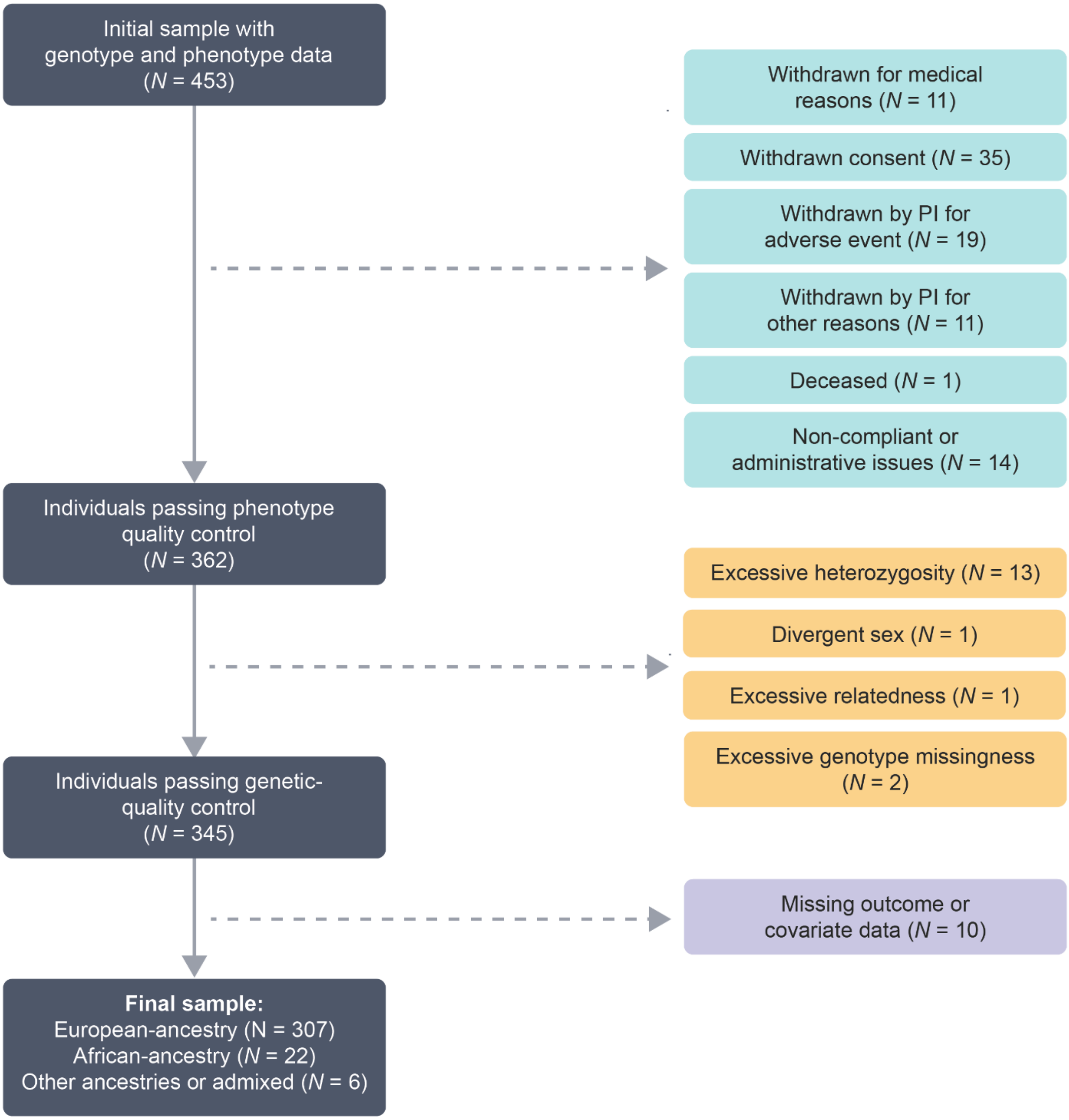

Supplementary Figure 1. Sample selection flow diagram.

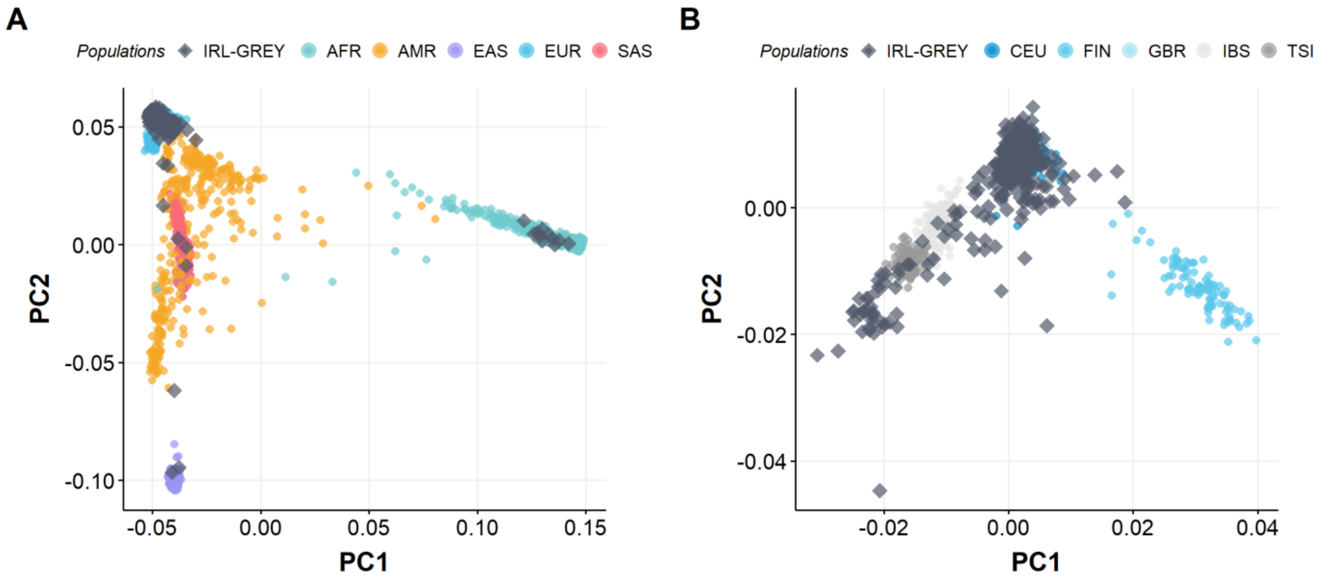

Supplementary Figure 2. Ancestry analysis.

Principal component analysis in the total, mixed-ancestry sample (*N*=345) and European sub-sample (*N*=307) across four extracted principal components.

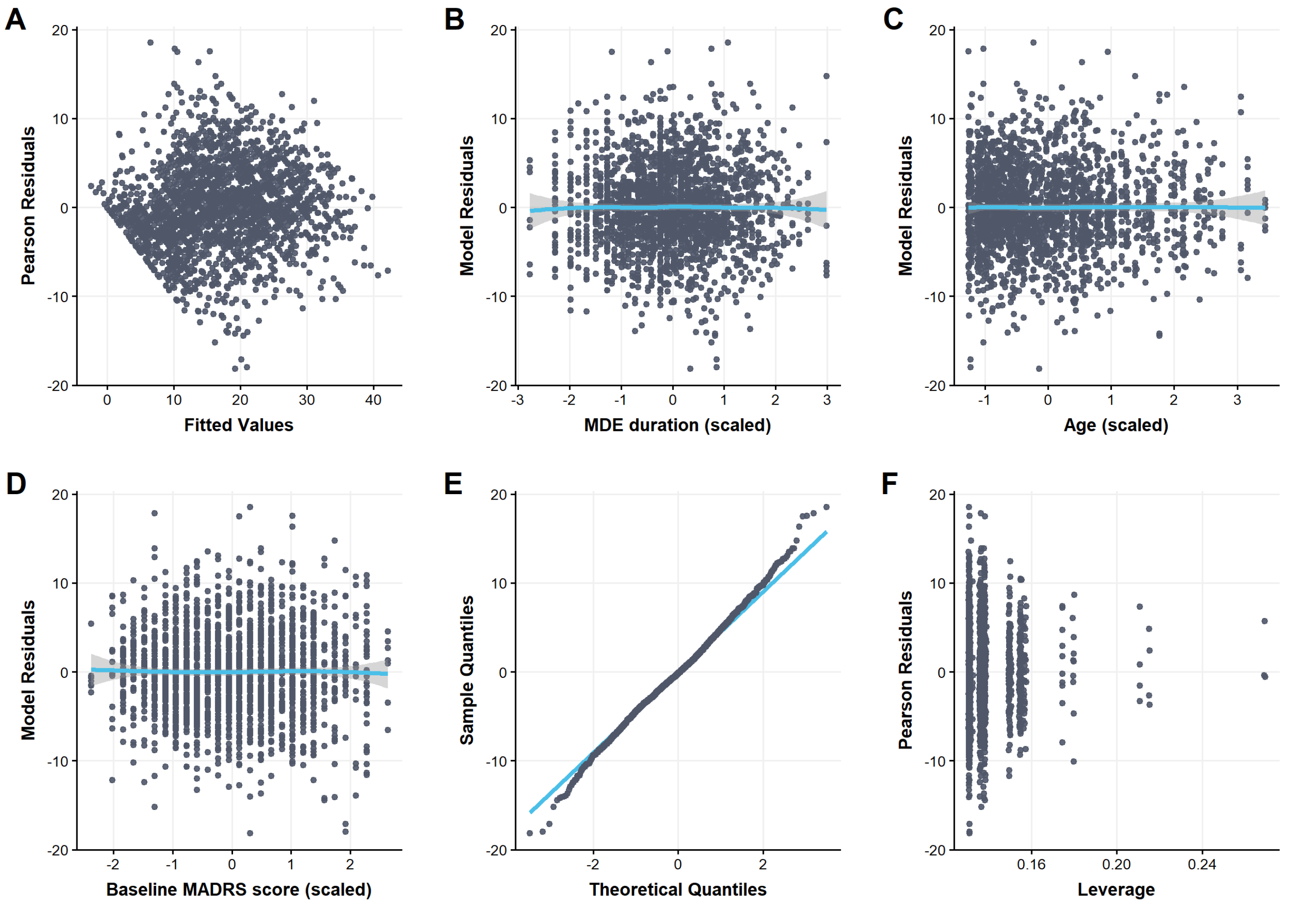

Supplementary Figure 3. Diagnostics of linear mixed-effects models.

Diagnostic plots are shown for rs6916777, including checks for linearity, homoscedasticity, and normality. Similar results were observed for rs12597726.

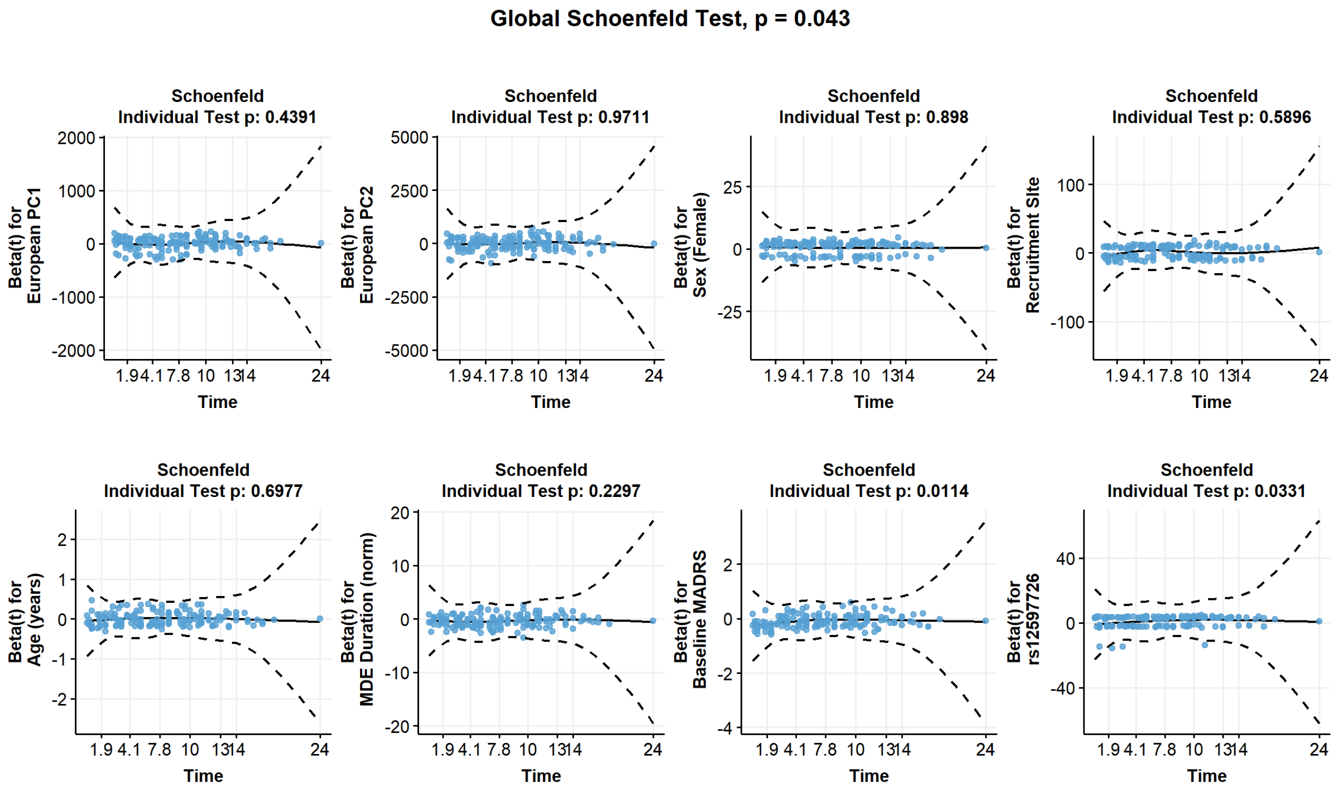

Supplementary Figure 4. Testing for proportional hazards.

Plots show scaled Schoenfeld residuals for all covariates included within the Cox proportional hazards model for rs12597726.

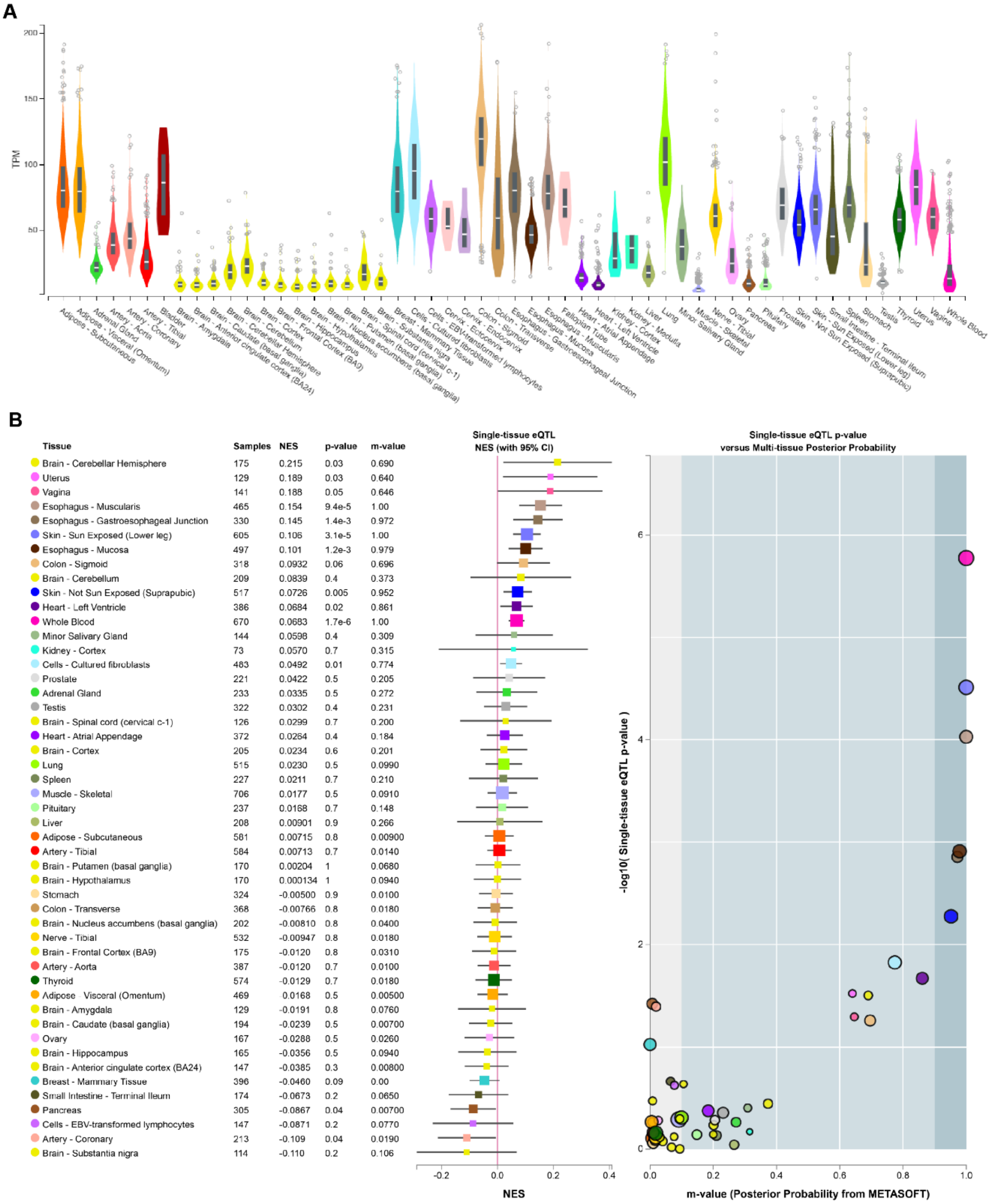

Supplementary Figure 5. GTEx expression information for *PIEZO1*.

(A) Expression *PEIZO1* across 54 GTEx tissues in transcripts per million (TPM; see https://www.gtexportal.org/home/gene/PIEZO1). **(B)** Effects of rs12597726 on the expression of *PIEZO1* across tissues where *NES* is the normalized effect size, with positive being an association of the minor allele with higher gene expression versus a negative value which would be decreased expression (see https://www.gtexportal.org/home/snp/rs12597726).

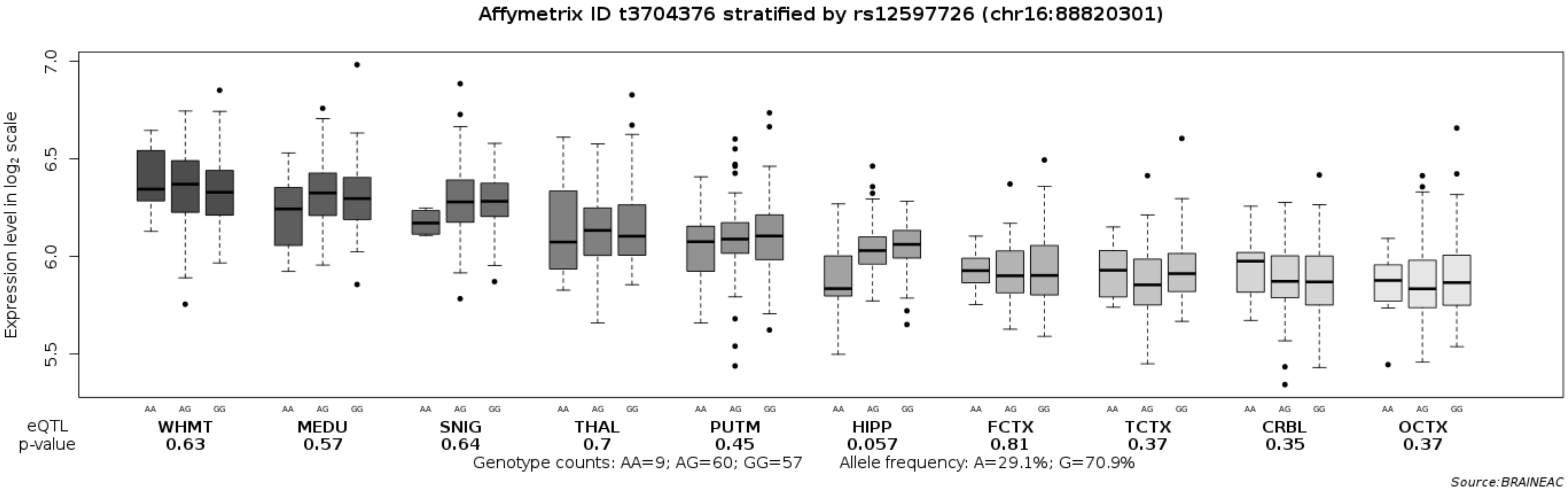

Supplementary Figure 6. Brain tissue expression of *PEIZO1*.

Expression levels are for the total transcripts across brain regions, stratified by rs12597726 genotypes, from *BRAINEAC*. *P*-values for eQTL below the names of brain regions compare levels of expression across genotypes.

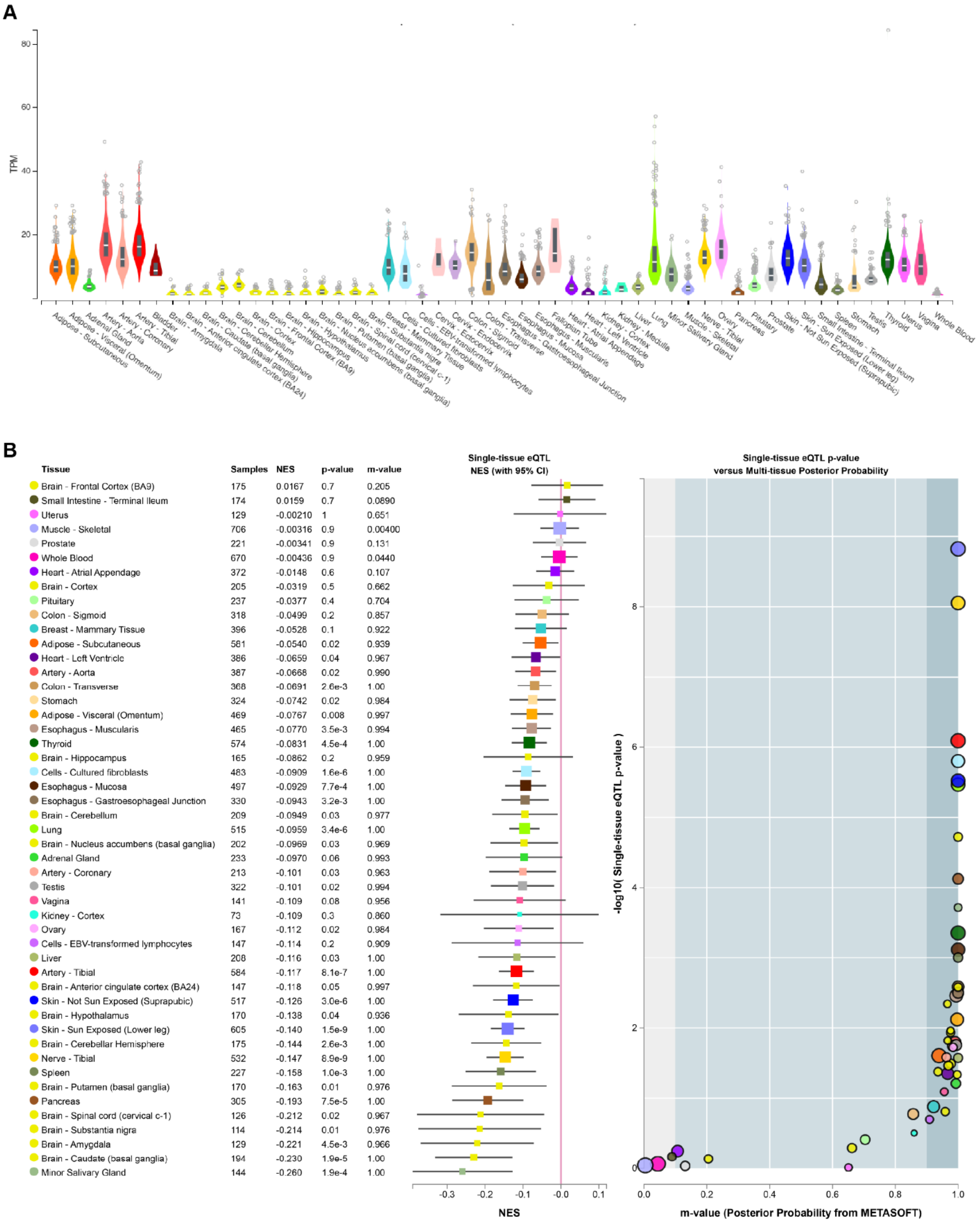

Supplementary Figure 7. GTEx expression information for *RNF217*.

(A) Expression *RNF217* across 54 GTEx tissues in transcripts per million (TPM; see https://www.gtexportal.org/home/gene/RNF217). **(B)** Effects of rs6916777 on the expression of *RNF217* across tissues where *NES* is the normalized effect size, with positive being an association of the minor allele with higher gene expression versus a negative value which would be decreased expression (see https://www.gtexportal.org/home/snp/rs6916777).

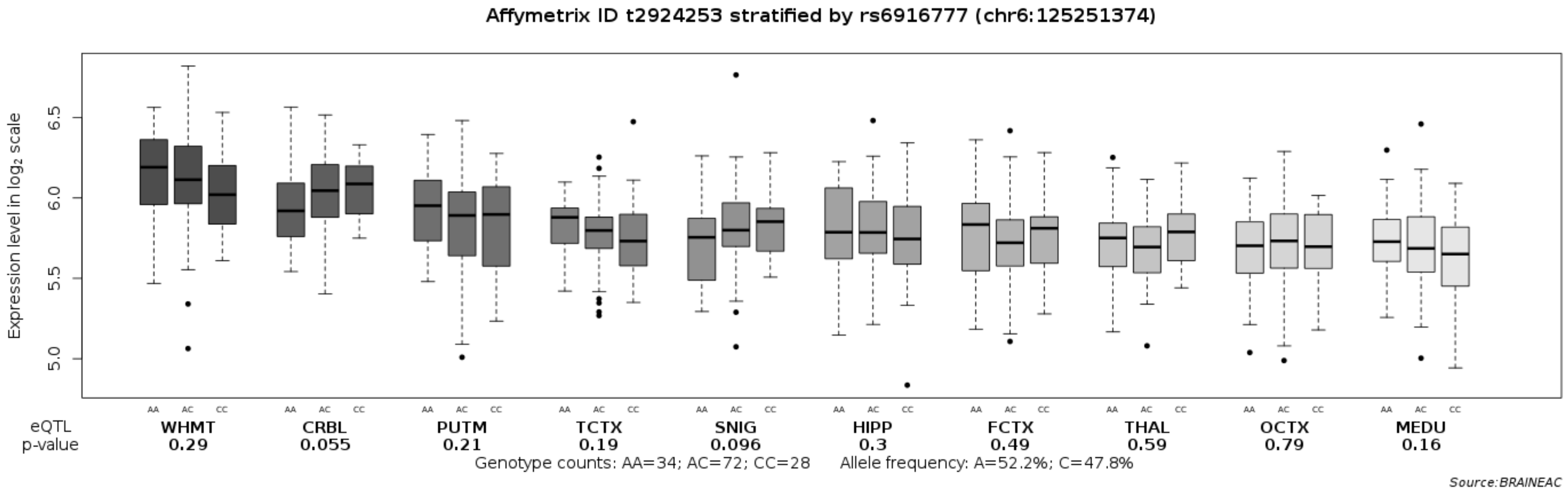

Supplementary Figure 8. Brain tissue expression of *RNF217.*

Expression levels are for the total transcripts across brain regions, stratified by rs12597726 genotypes, from *BRAINEAC*. *P*-values for eQTL below the names of brain regions compare levels of expression across genotype.
